# Genome-wide association analysis confirms and extends the association of S*LC2A9* with serum uric acid levels to Mexican Americans

**DOI:** 10.3389/fgene.2013.00279

**Published:** 2013-12-16

**Authors:** Venkata Saroja Voruganti, Jack W. Kent, Subrata Debnath, Shelley A. Cole, Karin Haack, Harald H. H. Göring, Melanie A. Carless, Joanne E. Curran, Matthew P. Johnson, Laura Almasy, Thomas D. Dyer, Jean W. MacCluer, Eric K. Moses, Hanna E. Abboud, Michael C. Mahaney, John Blangero, Anthony G. Comuzzie

**Affiliations:** ^1^Department of Genetics, Texas Biomedical Research InstituteSan Antonio, TX, USA; ^2^Department of Nutrition, Nutrition Research Institute, University of North Carolina at Chapel HillKannapolis, NC, USA; ^3^Division of Nephrology, Department of Medicine, University of Texas Health Science Center at San AntonioSan Antonio, TX, USA; ^4^Centre for Genetic Origins of Health and Disease, University of Western AustraliaPerth, WA, Australia

**Keywords:** variance components decomposition approach, joint linkage/association analysis, kinship, hyperuricemia

## Abstract

Increased serum uric acid (SUA) is a risk factor for gout and renal and cardiovascular disease (CVD). The purpose of this study was to identify genetic factors that affect the variation in SUA in 632 Mexican Americans participants of the San Antonio Family Heart Study (SAFHS). A genome-wide association (GWA) analysis was performed using the Illumina Human Hap 550K single nucleotide polymorphism (SNP) microarray. We used a linear regression-based association test under an additive model of allelic effect, while accounting for non-independence among family members via a kinship variance component. All analyses were performed in the software package SOLAR. SNPs rs6832439, rs13131257, and rs737267 in solute carrier protein 2 family, member 9 (*SLC2A9*) were associated with SUA at genome-wide significance (*p* < 1.3 × 10^−7^). The minor alleles of these SNPs had frequencies of 36.2, 36.2, and 38.2%, respectively, and were associated with decreasing SUA levels. All of these SNPs were located in introns 3–7 of *SLC2A9*, the location of the previously reported associations in European populations. When analyzed for association with cardiovascular-renal disease risk factors, conditional on *SLC2A9* SNPs strongly associated with SUA, significant associations were found for *SLC2A9* SNPs with BMI, body weight, and waist circumference (*p* < 1.4 × 10^−3^) and suggestive associations with albumin-creatinine ratio and total antioxidant status (TAS). The *SLC2A9* gene encodes an urate transporter that has considerable influence on variation in SUA. In addition to the primary association locus, suggestive evidence (*p* < 1.9 × 10^−6^) for joint linkage/association (JLA) was found at a previously-reported urate quantitative trait locus (Logarithm of odds score = 3.6) on 3p26.3. In summary, our GWAS extends and confirms the association of *SLC2A9* with SUA for the first time in a Mexican American cohort and also shows for the first time its association with cardiovascular-renal disease risk factors.

## Introduction

Hyperuricemia is a risk factor for gout, renal disease, and cardiovascular disease (CVD) (Cirillo et al., 2006; Nakagawa et al., 2006) and is known to aggregate in families (Dixon, 1960; Friedlander et al., 1988; Cameron and Simmonds, 2005). Variation in serum uric acid (SUA) levels is controlled by both genetic and environmental factors. Family-based studies have reported significant heritabilities for SUA levels with estimates ranging from 25 to 73% (Rao et al., 1982; Rice et al., 1990; Wilk et al., 2000; Tang et al., 2003; Yang et al., 2005; Nath et al., 2007; Voruganti et al., 2009a,b).

Genome-wide studies conducted to identify significant linkages for the variation in SUA have found quantitative trait loci (QTL) on several chromosomes for different populations; a QTL on chromosome 15 in Framingham Heart Study (Yang et al., 2005); on chromosome 8 in the Genetic Epidemiology Network of Arteriopathy (GENOA) (Rule et al., 2009); on chromosome 4p15 in an Australian Cohort (Cummings et al., 2010). However, none of them were in the same location as found previously in this Mexican American Cohort (Voruganti et al., 2009a). Similarly, genome-wide association (GWA) and candidate gene studies have found several genes to be associated with SUA, mainly solute carrier protein 2 family, member 9 *(SLC2A9*) (Li et al., 2007; Brandstätter et al., 2008; Caulfield et al., 2008; Dehghan et al., 2008; Döring et al., 2008; McArdle et al., 2008; Stark et al., 2008; Vitart et al., 2008; Wallace et al., 2008; Kolz et al., 2009; Charles et al., 2011; Karns et al., 2012), ATP-binding cassette, subfamily G, member 2 (*ABCG2*) (Kolz et al., 2009; Woodward et al., 2009; Nakayama et al., 2011; Karns et al., 2012; Zhang et al., 2013); solute carrier protein 22 family, members 11 and 12 (*SLC22A11*, *SLC22A12*) (Kolz et al., 2009; Tin et al., 2011), solute carrier protein 16 family, member 9 *(SLC16A9*), PDZ domain containing 1(*PDZK1*) (Kolz et al., 2009). A large GWAS in another European cohort, Köttgen et al. (2013), confirmed or replicated 28 previously reported associations for SUA. However, most of these studies were conducted in Caucasian, African American, or Asian populations. To date no GWAS of SUA levels have been conducted in Mexican Americans—a population affected with disproportionate burden of CVD and type 2 diabetes (Flegal et al., 1991; Go, 2013; http://www.diabetes.org/diabetes-basics/diabetes-statistics/).

SUA is a strong marker of risk for renal disease and is considered clinically relevant and of high significance. Its role as a major risk factor in the development of kidney stones or nephrolithiasis and gout is well-recognized. The relationship between uric acid and kidney function seems to be two-sided. On one hand, decline in glomerular filtration rate (GFR) (kidney function parameter) may lead to elevation of uric acid, on the other hand, increase in uric acid seems to alter glomerular function through renal vasoconstriction and increased rennin expression (Sánchez-Lozada et al., 2002). Hyperuricemia is also known to induce endothelial dysfunction and inflammation indicating a role in atherosclerosis as well as chronic kidney disease (Ejaz et al., 2012). Rodent Sánchez-Lozada et al., 2002; Ejaz et al., 2012 and human studies (Nakagawa et al., 2005; Liebman et al., 2007) have shown the role of hyperuricemia in hypertension, atherosclerosis, CVD, initiation and progression of renal disease, and metabolic syndrome. In fact, drugs that decrease SUA levels, mainly allopurinol, have been shown to improve survival in chronic heart failure patients (Gotsman et al., 2012), improve endothelial function in patients with chronic kidney disease (Yelken et al., 2012), and reduce oxidative stress and improve endothelial function in patients with coronary artery disease (Rajendra et al., 2011). Elaborating on the protective effects of reduced SUA levels on end-stage renal disease (ESRD), Doria and Krolewski (2011) point out that lowering of SUA may be a potential approach to treat ESRD in patients with diabetes.

Here we present results of the first GWAS in Mexican Americans of the San Antonio Family Heart Study (SAFHS) that attempts to identify genetic factors that affect the variation in SUA. We extend these findings to the analysis of association of *SLC2A9* with cardiovascular and renal factors given the role of SLC2A9 in hypertension and renal urate transport.

## Materials and methods

Population characteristics: The San Antonio Family Heart Study (SAFHS) was initiated in 1991 to identify genes influencing the risk of CVD in Mexican Americans. Study subjects have been recalled up to three times to acquire longitudinal data; the analysis reported here is based on the second recall, 2002–2006. Individuals were recruited from large Mexican American families, residing in San Antonio, TX without regard to disease status. Participants in this study were recruited from 40 extended families, with probands between 40 and 60 years of age (MacCluer et al., 1999; Mitchell et al., 1999). Eligibility criteria required the proband to have at least six first-degree relatives (excluding their parents) 16 years or older and who resided in San Antonio, TX. At each recruitment phase, subjects were brought to a research clinic at the University of Texas Health Science Center—San Antonio (UTHSCSA) for interview and examination by trained recruiters and nurses. Anthropometrics including height, weight, and waist circumference; blood pressure; and self-reported information regarding medical history and socio-demographic status were obtained in all phases of SAFHS data collection. Blood samples were collected from all participants after an overnight fast and plasma and serum were prepared and stored at −80°C until analyzed. Blood samples were also drawn at 2 h after a standard oral glucose tolerance test; diabetes was diagnosed if the 2 h glucose level was 11.1 mmol/l or higher, or if the subject had been prescribed antidiabetic medication. The final analysis sample with complete phenotype and genotype data included 632 SAFHS participants. Written informed consent was obtained from all participants to participate in this study. All research and consenting protocols were approved by the Institutional Review Board of the UTHSCSA.

### Phenotyping

Uric acid was measured in serum by a colorimetric assay using uricase and peroxidase (Domagk and Schlicke, 1968). Serum creatinine was estimated by the modified kinetic Jaffe reaction (Beckman Synchron LX System). GFR was estimated by the MDRD equation: eGFR (ml/min/1.73 m2 body surface area) = 186 × serum creatinine × age × (−0.203) × (0.742 if female) × (1.210 if black) (Arar et al., 2008). Cardiovascular risk factors included blood pressure, weight, waist circumference as well as fasting plasma levels of glucose, insulin, lipids, and cytokines measured by standardized reference procedures (Arar et al., 2008; Voruganti et al., 2009a). A single-void morning urine sample was collected from each participant for measuring albumin and creatinine and estimating albumin to creatinine ratio (UACR). Description of their measurement techniques are given in Arar et al. (2008). Indicator variables were coded for diabetes diagnosis and for data from self-report and medical history on current use of blood pressure medication, alcohol consumption, and smoking. Covariates of SUA were included in analysis models as described below and in the Results.

### Genome-wide association (GWA) analysis

GWA analysis was conducted in the SAFHS based on SNP genotypes obtained using the Illumina HumanHap550 BeadChip (Illumina, San Diego, CA). Our experimental error rate (based on duplicates) was 2 per 100,000 genotypes. The average call rate per individual sample was 97%. Approximately 1 per 1000 genotypes was blanked due to Mendelian errors. Specific SNPs were removed from analysis if they had call rates <95% (about 4000 SNPs) or deviated from Hardy–Weinberg equilibrium at 5% false discovery rate (FDR) (12 SNPs). Missing genotypes were imputed from pedigree data using MERLIN (Abecasis et al., 2002). SNP genotypes were checked for Mendelian consistency using the program SimWalk2(Sobel and Lange, 1996). The estimates of the allele frequencies and their standard errors were obtained using SOLAR (Almasy and Blangero, 1998).

### Measured genotype analysis

Each SNP genotype was converted in MERLIN (Abecasis et al., 2002) to a covariate measure equal to 0, 1, or 2 copies of the minor allele (or, for missing genotypes, the weighted covariate based on imputation). These covariates were included in the variance-components mixed models for measured genotype analyses (MGA; Boerwinkle et al., 1986) vs. null models that incorporated the random effect of kinship. For the initial GWA screen, SUA levels were regressed on selected covariates (see Results) and the association of the inverse-normalized residuals with each SNP covariate was tested independently as a 1 degree of freedom likelihood ratio test. Empirical thresholds for genome-wide significant and suggestive evidence of association were based on the distribution of *p*-values from 10,000 simulated null GWAS (i.e., simulations of a heritable trait with no modeled SNP covariate effects using the SAFHS pedigree and genotypes). The threshold for significance (*p* < 1.3 × 10^−7^) was defined as the cutoff for the lower 5% tails of the empirical distribution, and the threshold for suggestive evidence (*p* < 1.6 × 10^−6^) was the minimum *p*-value obtained not more than once per genome scan.

### Linkage and joint linkage/association analysis

Multipoint linkage analysis (Almasy and Blangero, 1998) was performed in SOLAR using estimates of locus-specific allele sharing based on genotypes for 461 STR markers. In addition, we employed a novel joint linkage/association (JLA) analysis for each SNP that tested each saturated model (including linkage and the fixed effect of the SNP) against a null model in which both effects were constrained to zero. Regression parameters for selected covariates (see Results) were estimated simultaneously with the linkage and association parameters. Because the linkage variance parameter was tested on its lower boundary, the distribution of the likelihood ratio test statistic is distributed as a 1:1 mixture of chi-square distributions with 1 and 2 degrees of freedom, respectively (Self and Liang, 1987).

### Analysis of cardiovascular and renal disease risk factors for association with *SLC2A9* SNPs

**Figure 1:**
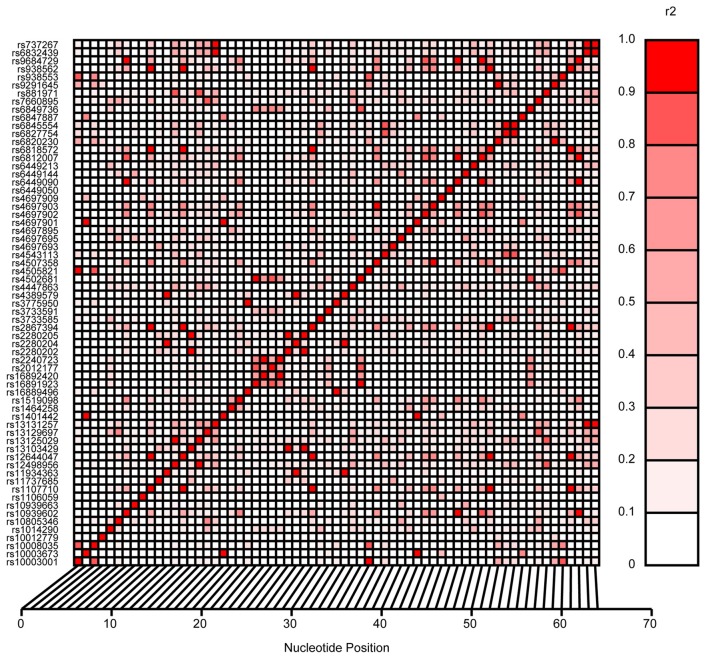
**Linkage disequilibrium pattern of *SLC2A9* SNPs investigated in this study**.

Cardiovascular and renal disease risk factors that were included in the analysis were anthropometric measures such as body weight, BMI, waist circumference, circulating levels of lipids such as triglycerides, high, low-density lipoprotein and total cholesterol, glucose, insulin, and kidney function phenotypes (serum creatinine, eGFR, and UACR). For these analyses, the appropriate significance level was determined to be 1.4 × 10^−3^ based on the number of SNPs investigated in *SLC2A9*. This significance value was computed taking into account the linkage disequilibrium (LD) pattern of these SNPs (Figure 1).

## Results

The mean ± SE age and SUA levels of participating individuals (*n* = 632) were 47.87 ± 14.8 yrs and 5.35 ± 1.38 mg/dl, respectively, with men having higher levels of SUA than women (6.1 ± 1.7 vs. 4.94 ± 1.6 mg/dl). Significant heritability was detected for SUA levels (*h*^2^ = 0.39, *p* = 2.3 × 10^−8^) with age, sex, age^2^, interaction between age and sex, waist circumference, systolic blood pressure, plasma triglyceride, plasma high-density lipoprotein, serum creatinine, and indicator variables for use of blood pressure medication, alcohol use, smoking status, and diabetic status as covariates.

### Genome-wide linkage/association analysis

**Figure 2:**
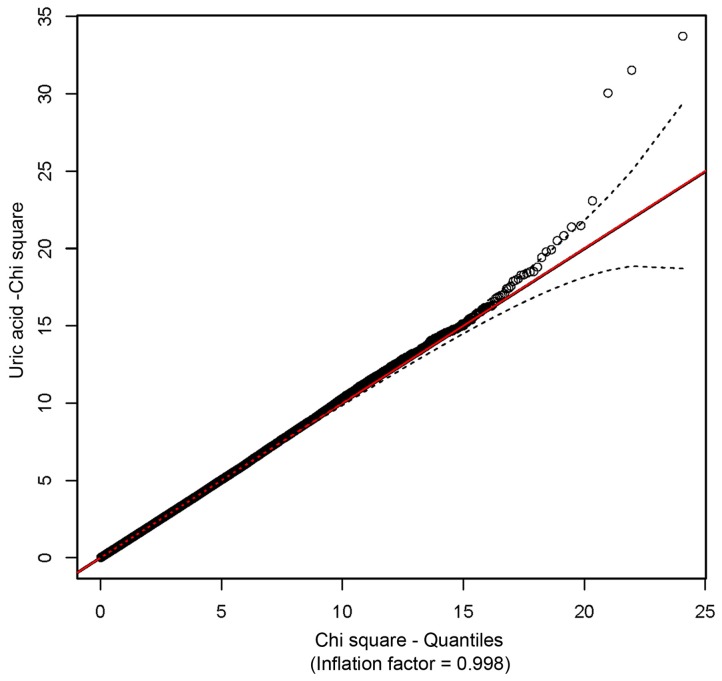
**A Q-Q plot showing the absence of inflation due to population stratification**.

Association and JLA tests were performed using a custom script for SOLAR (Almasy and Blangero, 1998) as described in methods. All models included the random kinship as well as fixed effects of SNPs. Prior to analysis, SUA levels were regressed on age, sex, age^2^, interaction between age and sex, waist circumference, systolic blood pressure, plasma triglyceride, plasma high-density lipoprotein, serum creatinine, blood pressure medication, alcohol intake, smoking status and diabetic status, and the residuals were inverse-normal transformed. The distribution of *p*-values from GWAS of SUA showed no evidence of inflation due to population stratification (Figure 2).

**Figure 3:**
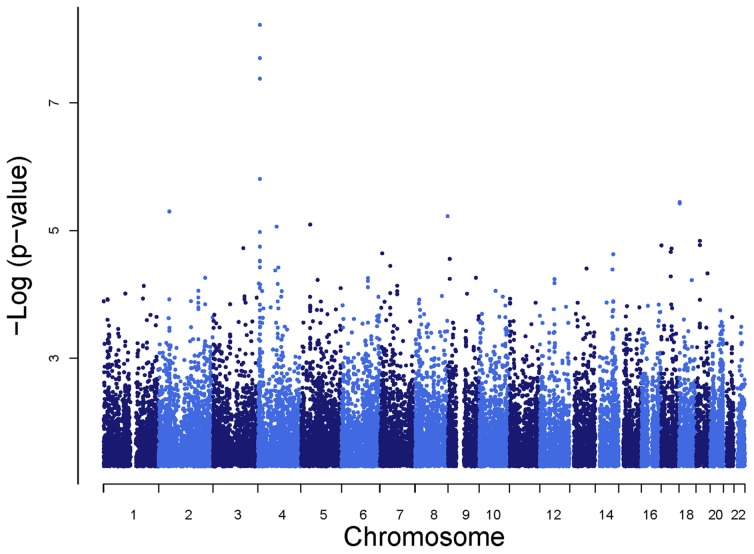
**Genome-wide association analysis of serum uric acid**.

**Figure 4:**
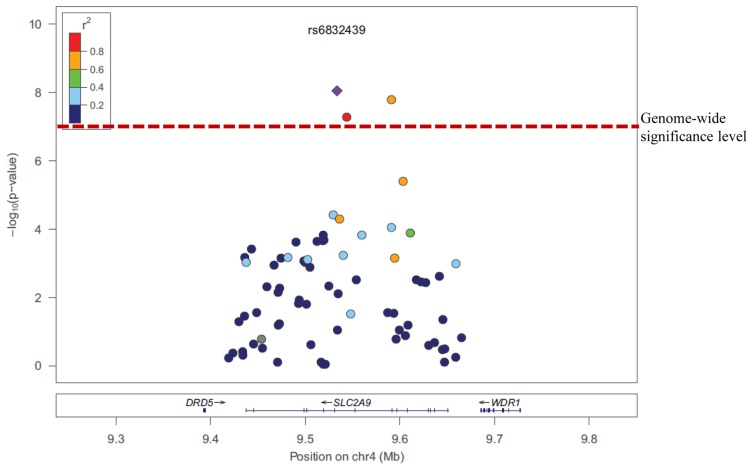
**Association of serum uric acid with *SLC2A9* SNPs**.

**Figure 5:**
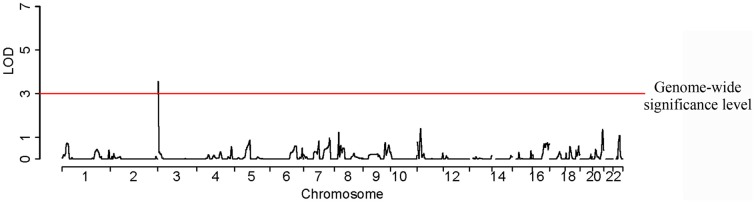
**A joint linkage-association approach shows a significant LOD score for serum uric acid on chromosome 3**.

SNPs rs6832439, rs13131257, and rs737267 in *SLC2A9* were associated with SUA levels at genome-wide significance (*p* < 1.3 × 10^−7^) (Table 1, Figures 3, 4). The minor alleles of these SNPs had frequencies of 0.36, 0.36, and 0.38, respectively, and were associated with decreasing SUA levels. One additional SNP rs6449213 showed suggestive association (*p* < 1.6 × 10^−6^) with SUA levels. All these SNPs are located in introns 3–7 of *SLC2A9.* The allele frequencies of SNPs that had significant association with SUA are shown in Table 1. Minor alleles of the main four SNPs were associated with lower SUA levels (Table 2). We had previously reported significant evidence of linkage for SUA (*LOD* = 3.5) on chromosome 3p (Voruganti et al., 2009a). Using the JLA test, there was suggestive evidence of association of SUA with SNPs in *CNTN4*, a gene within the 1-LOD support interval of our peak linkage signal (Table 1, Figure 5).

**Table 1:** **Joint linkage-association analysis of serum uric acid (Empirical genome-wide significance: *p* < 1.3 × 10^−7^)**.

**Gene**	**SNP^a^**	**Coordinates (bp)**	**MGA^b^ (*p*-value)**	**JLA^c^ (*p*-value)**	**Minor allele/frequency**
*SLC2A9*^d^	rs6832439	9924319	6.0 × 10^−9^	2.7 × 10^−8^	A/0.36
*SLC2A9*	rs13131257	9981889	2.0 × 10^−8^	8.1 × 10^−8^	T/0.36
*SLC2A9*	rs737267	9934744	4.2 × 10^−8^	1.7 × 10^−7^	A/0.38
*SLC2A9*	rs6449213	9994215	1.6 × 10^−6^	5.7 × 10^−6^	C/0.22
*CNTN4*^e^	rs7652782	2821616	3.4 × 10^−4^	7.1 × 10^−7^	A/0.06
*CNTN4*	rs6786387	2822150	2.6 × 10^−4^	7.6 × 10^−7^	A/0.03
*CNTN4*	rs6786174	2982630	6.1 × 10^−4^	1.2 × 10^−6^	C/0.08
*CNTN4*	rs17586876	2964182	1.3 × 10^−3^	2.0 × 10^−6^	G/0.09

a SNP, Single Nucleotide Polymorphism.

b MGA, Measured Genotype Analysis.

c JLA, Joint linkage association analysis.

d SLC2A9: solute carrier protein 2 family, member 9.

e CNTN4: Contactin 4.

**Table 2:** **Association of the most significant SNPs in solute carrier protein 2 family, member 9 (*SLC2A9*) gene with serum uric acid (mg/dl)**.

**SNP**	**Mean effect size (%)^a^**	**11^b^**	**12**	**22**
rs6832439	5.3	5.6 (1.4)^c^	5.3 (1.4)	4.8 (1.3)
rs13131257	4.9	5.6 (1.4)	5.3 (1.4)	4.8 (1.4)
rs737267	5.0	5.6 (1.4)	5.3 (1.4)	4.8 (1.3)
rs6449213	4.5	5.5 (1.4)	5.2 (1.4)	4.2 (1.2)

a Proportion of the residual phenotypic variance that is explained by the minor allele of the SNP.

b 1 – major allele; 2 – minor allele.

c genotype-specific mean (standard deviation) of serum uric acid levels (mg/dl).

### Association of *SLC2A9* SNPs with cardiovascular and renal disease risk factors

In addition to SUA, we also tested the association of *SLC2A9* SNPs with several renal and CVD-related risk factors such as anthropometrics, glucose, insulin, lipids, cytokines, and renal function phenotypes. Of these, SNPs in the *SLC2A9* gene were significantly associated with body weight, BMI, waist circumference, total antioxidant status (TAS) and urinary albumin-creatinine ratio (UACR) (Table 3). However, the SNPs associated with SUA levels were not the same as those associated with body weight, BMI, waist circumference, or ACR. Interestingly, the two SNPs associated with TAS were also associated with SUA levels.

**Table 3:** **Solute carrier protein 2 family, member 9 (*SLC2A9*) SNPs (chromosome 4) associated with cardiovascular or renal disease risk factors**.

**Trait**	**SNP^b^**	**MGA^c^ (*p*-value)**	**SNP coordinates**	**Minor/major allele**	**Minor allele frequency**	**Effect size(%)^d^**
Body weight	rs938553	5.5 × 10^−6^	9925526	A/G	0.12	2.8
BMI	rs10003001	9.8 × 10^−4^	9984475	A/G	0.07	2.1
	rs10008035	9.1 × 10^−4^	9999335	A/C	0.13	2.0
	rs938553	7.3 × 10^−4^	9925526	A/G	0.12	2.3
Waist circumference	rs10003001	5.0 × 10^−4^	9984475	A/G	0.07	2.2
	rs10008035	8.0 × 10^−4^	9999335	A/C	0.13	2.0
	rs938553	2.0 × 10^−4^	9925526	A/G	0.12	2.6
Albumin/creatinine ratio	rs1014290	4.0 × 10^−4^	10001861	G/A	0.33	2.1
	rs10805346	6.0 × 10^−4^	9920347	A/G	0.41	2.0
	rs13129697	3.0 × 10^−4^	9926967	C/A	0.48	2.2
	rs7660895	6.0 × 10^−4^	9985445	A/G	0.46	2.0
Total antioxidant status	rs6832439	1.4 × 10^−6^	9924319	A/G	0.36	2.9
	rs737267	1.4 × 10^−6^	9934744	A/C	0.38	2.9

^a^Empirical significance based on number of SNPs analyzed in SLC2A9 gene (p < 1.4 × 10^*−3*^).

b SNP, Single Nucleotide Polymorphism.

c MGA, Measured Genotype Analysis.

d Proportion of the residual phenotypic variance that is explained by the minor allele of the SNP.

When association was conducted, conditional on SNPs associated with SUA, *SLC2A9* SNPs showed evidence of significant association with BMI, waist circumference and body weight, and suggestive association with TAS and UACR (data not shown).

## Discussion

Our GWAS found polymorphisms in *SLC2A9* to be significantly associated with SUA levels in Mexican Americans. This is the first GWAS for SUA in Mexican Americans and replicates results from several studies conducted in European populations from the UK, Germany, Croatia and Sardinia. Recent GWAS have shown consistently that single nucleotide polymorphisms (SNPs) in the *SLC2A9* gene are associated with SUA levels (Li et al., 2007; Döring et al., 2008; McArdle et al., 2008; Stark et al., 2008; Vitart et al., 2008; Zemunik et al., 2009; Yang et al., 2010). A study conducted in a Croatian sample found that *SLC2A9* variants were associated with SUA with SNPs explaining 1.7–5.3% of the variance in SUA levels. In the same study, this association was replicated in a sample from the island of Orkney (Vitart et al., 2008). Two of the three SNPs associated with SUA in this study were the same as shown in our study (rs6449213 and rs737267). In addition, the effect sizes (proportion of residual phenotypic variance explained by the SNP) of our significant association are in the same range (4–5% of total phenotypic variance). Similarly, in a cohort from Germany, SUA levels were associated with *SLC2A9* variants (19). Of the several SNPs that were associated only rs6449213 was common between our two studies. They also showed sex-specific effects of variants in *SLC2A9* on SUA levels. This finding was replicated by Brandstätter et al. (2008). Other studies that showed association of polymorphisms in *SLC2A9* with SUA were conducted in individuals from Sardinia (Li et al., 2007), Germany (Stark et al., 2008), and the United Kingdom (Wallace et al., 2008; Kolz et al., 2009) as well as in Asian (Matsuo et al., 2008; Cummings et al., 2010; Tabara et al., 2010; Tu et al., 2010; Guan et al., 2011) and African American populations (Dehghan et al., 2008; Rule et al., 2011). To investigate the functional aspects of the *SLC2A9* gene, Caulfield et al. (2008) showed that SLC2A9 was a high-capacity urate transporter and can exchange glucose for urate in the process of secretion of urate into the urine. They also confirmed the previously reported association of *SLC2A9* with SUA in six different cohorts of European ancestry. Several other loci have also been reported to be associated with serum uric acid levels in GWAS as well as candidate gene association studies (Table 4).

**Table 4:** **Previously reported association of serum uric acid levels with genes other than *SLC2A9***.

**SNP**	**Joint linkage/association (JLA) in our study (*p*-value)**	**Gene name**	**Gene symbol**	**Population**	**References**
rs2231142	0.24^a^	ATP-binding cassette family G, member 2	*ABCG2*	Mexican Americans, African Americans, European Americans, American Indians	Köttgen et al., 2013; Zhang et al., 2013
				European Americans	Kolz et al., 2009
				Japanese	Tabara et al., 2010
				Whites and Blacks	Dehghan et al., 2008
rs1471633	0.79 (rs1967017)^b^	PDZ domain containing 1	*PDZK1*	European Americans	Köttgen et al., 2013
rs12129861	0.17 (rs1298954)^c^	PDZ domain containing 1	*PDZK1*	European Americans	Kolz et al., 2009
rs1171614	0.11 (rs1171617)^b^	Solute carrier protein family 16A, member 9	*SLC16A9*	European Americans	Kolz et al., 2009; Köttgen et al., 2013
rs12800450	0.05 (rs505802)^c^	Solute carrier protein family 22A, member 12	*SLC22A12*	African Americans	Tin et al., 2011
rs2078067	0.02 (rs10792438)^c^	Solute carrier protein family 22A, member 11	*SLC22A11*	European ancestry	Yang et al., 2010
rs1165205	0.92 (rs9393672)^b^	Solute carrier protein family 17A, member 3	*SLC17A3*	Whites and Blacks	Dehghan et al., 2008

a SNP typed on our array.

b Proxy SNP on our array.

c No proxy SNP available; strongest evidence of JLA in our study annotated to this gene.

Proxy SNPs identified with SNAP (SNP Annotation and Proxy Search) Version 2.2: https://www.broadinstitute.org/mpg/snap/

The solute carrier protein 2 family, member 9 *(SLC2A9*) gene was recently cloned and identified as a member of the solute carrier protein 2 family (Le et al., 2008). Originally thought to be only a facilitated hexose transporter, it was found to be primarily involved in uric acid transport. Its two forms SLC2A9a and SLC2A9b are expressed in the basolateral and apical membranes of the kidney while SLC2A9a is also expressed in liver. Their amino acid sequences are identical, except that SLC2A9b has a shorter and modified N-terminus (Preitner et al., 2009). Both forms are involved in renal urate transport (Le et al., 2008; Cheeseman, 2009). Caulfield et al. (2008) proposed that extracellular glucose and intracellular urate are exchanged by the SLC2A9 transporter. In the kidney SLC2A9 maintains uric acid reabsorption independent of other uric acid transporters (Preitner et al., 2009). On one hand *SLC2A9* mutations are associated with hypouricemia and hyperuricosuria, while on the other, mutations in *SLC2A9* that alter the protein impair urate secretion into the urine resulting in hyperuricemia (Le et al., 2008; Cheeseman, 2009).

Given the ubiquity of evidence for association of SUA with *SLC2A9* polymorphisms and the relatively large effect size of these associations reported in other studies, it is not surprising that we could identify this locus in our study despite its modest sample size. In addition, our joint-linkage association approach has the potential to maximize the information in a sample of related individuals, thus amplifying a signal taking into account fixed effects of marker genotypes (association) and the random effects of shared sequence identity (linkage). With this approach we found suggestive evidence of association on chromosome 3p25-p26 as we showed in our prior linkage analysis (Voruganti et al., 2009a). Associated SNPs were found within the contactin 4 (*CNTN4*) gene, which is located in the one-LOD support interval in our previous linkage scan for SUA levels. The *CNTN4* gene codes for a member of the contactin subgroup of cell adhesion molecules of the immunoglobulin (Ig) superfamily. This family has an important role in the formation and functioning of the neuronal networks; specifically, CNTN4 is known to play a key role in development of the central nervous system. The possible functional relationship of this gene to SUA levels is unknown.

Our association and JLA results are especially interesting given the recent attention to the relative importance of common and rare genetic variants for risk of common complex diseases (Maher, 2008; Manolio et al., 2009). Common marker variants like those in *SLC2A9* (minor allele frequency 36–38% in our cohort) should, in theory, be in LD with one or more common *functional* variants whose effect on the trait of interest should be similar in different lineages. On the other hand, low-frequency functional variants (and low-frequency marker alleles in LD with them) are expected to appear in only a subset of lineages; these pedigree-specific effects are more likely to be identified by linkage than by association. In this context, it is interesting that our replication of association at *SLC2A9* is not matched by a linkage signal, while the SNPs identified by joint linkage and association within a strong linkage peak on chromosome 3 have low minor allele frequencies (3–9%)—both findings being consistent with theoretical expectation. These results may also explain why our chromosome 3 QTL has apparently not been reported by other studies, which are primarily population-based; however, the current upsurge in analysis of next-generation sequencing data and improved methods for rare variant detection may provide opportunities to test our findings in the near future.

Since SUA is associated with obesity, type 2 diabetes and CVD risk and is advocated by many to be included as a component of the metabolic syndrome, we conducted a measured genotype analysis for *SLC2A9* SNPs and these risk factors. We found evidence of significant associations of *SLC2A9* SNPs with body weight, BMI and waist circumference and suggestive associations with TAS. In the same study we also reported significant genetic correlations between SUA and waist circumference in the same study (Voruganti et al., 2009a). Variants in the uric acid transporter gene (*SLC2A9*) that have been associated with lower SUA were also associated with decreased blood pressure (Parsa et al., 2012). Similarly, another *SLC2A9* variant (rs1014290), associated with lower SUA were also associated with decreased risk for diabetes mellitus (Liu et al., 2011) in Han Chinese. In a study by Brandstätter et al. (2008), the association between *SLC2A9* SNPs and SUA was significantly modified by BMI. They showed stronger effect size in individuals with higher BMI. However, the precise mechanism that may explain this association is not clear. In contrast, Vitart et al. (2008) found no association between *SLC2A9* SNPs and metabolic syndrome components. Caulfield et al. (2008) explored the association of *SLC2A9* SNPs and blood pressure traits and found no significant association. We also observed that the SNPs associated with body weight, BMI, or waist circumference were different than those associated with SUA levels. However, the two SNPs associated with TAS were also associated with SUA levels. SUA is known to act as a free radical scavenger (Pasalic et al., 2012) and combat oxidative stress (Johnson et al., 2011). Therefore, the possible link between them may be the role of SUA as an “antioxidant.”

In addition, we found suggestive association between *SLC2A9* SNPs and the renal disease development marker UACR. Knock out rodent studies have shown that *SLC2A9* knockout mice develop urate nephropathy characterized by intra-tubular urate lithiasis, interstitial inflammation and fibrosis and tubular atrophy (Preitner et al., 2009). SUA levels in humans are primarily determined by renal uric acid clearance, with two-thirds of uric acid turnover being accounted for by its urinary excretion. Hyperuricemia is predictably associated with a decrease in GFR (Culleton, 2001; Johnson et al., 2003; Feig, 2009) and 90% of clinically-recognized hyperuricemia results from impaired renal excretion of uric acid (Le et al., 2008). Abnormal renal urate handling can promote both uric acid and calcium oxalate nephrolithiasis by distinct mechanisms; low urine pH favors uric acid crystallization while mixed uric acid- or sodium urate-calcium oxalate stones develop by epitaxy, “salting out” of calcium oxalate or adsorption of crystal growth inhibitors (Liebman et al., 2007). Hyperuricemia has also been implicated in the development of hypertension, a major risk factor for the development and progression of chronic kidney disease (Feig, 2009). Micropuncture studies have shown that elevated uric acid levels cause cortical vasoconstriction and increase glomerular capillary pressure leading to progressive glomerular injury (Sánchez-Lozada et al., 2002). Given the role of SLC2A9 in renal urate transport, the association between *SLC2A9* SNPs and UACR assumes pathogenic significance.

To summarize, this is the first study to report the association of polymorphisms in the *SLC2A9* gene with SUA levels in a genome-wide analyses in Mexican Americans. In addition, this is first time that any association between *SLC2A9* SNPs and BMI, body weight, waist circumference, UACR, and TAS has been reported.

## Author contributions

Conceived and designed the experiments: Venkata Saroja Voruganti, Jack W. Kent Jr., John Blangero, Anthony G. Comuzzie; Performed or supervised all aspects of statistical analyses: Venkata Saroja Voruganti, Jack W. Kent Jr., John Blangero, Anthony G. Comuzzie; Helped with statistical analyses: Harald H. H.Göring, Laura Almasy, Thomas D. Dyer, Michael C. Mahaney, John Blangero, Anthony G. Comuzzie; Helped with preparation/editing of manuscript: Subrata Debnath, Shelley A. Cole, Karin Haack, Harald H. H.Göring, Melanie A. Carless, Joanne E. Curran, Matthew P. Johnson, Laura Almasy, Thomas D. Dyer, Jean W. MacCluer, Eric K. Moses, Hanna E. Abboud, Michael C. Mahaney, John Blangero, Anthony G. Comuzzie; Wrote the manuscript: Venkata Saroja Voruganti, Jack W. Kent Jr.

### Conflict of interest statement

The authors declare that the research was conducted in the absence of any commercial or financial relationships that could be construed as a potential conflict of interest.
